# Epstein–Barr Virus Load in the Saliva of Patients with Oropharyngeal Cancer—Could It Have Prognostic Significance?

**DOI:** 10.3390/v17111523

**Published:** 2025-11-20

**Authors:** Karol Paradowski, Magdalena Góralczyk, Bartłomiej Drop, Mirosław Jarosz, Małgorzata Polz-Dacewicz

**Affiliations:** 1 Aldent—Center for Dentistry and Implantology, 20-631 Lublin, Poland; karol.paradowski@aldent.lublin.pl; 2 Department of Virology with Viral Diagnostics Laboratory, Medical University of Lublin, 20-093 Lublin, Poland; magdalena.goralczyk@umlub.edu.pl; 3 Department of Computer Science and Medical Statistics with the e-Health Laboratory, 20-090 Lublin, Poland; bartlomiej.drop@umlub.edu.pl; 4 Faculty of Human Sciences, University of Economics and Innovation, 20-209 Lublin, Poland; miroslaw.jarosz@wsei.pl

**Keywords:** oropharyngeal cancer, saliva, EBV DNA load, anti-EBV antibodies, biomarker MMP3, MMP9, NFκB

## Abstract

The discovery of EBV over 60 years ago was a breakthrough in understanding the development of many cancers, including head and neck cancers, but many processes remain to be explained. Scientists, looking for new biomarkers, have recently been paying a lot of attention to salivary EBV DNA load. Oral EBV DNA load may indicate not only EBV lytic replication activity but also potentially correlate with EBV-related disease progression. Numerous studies indicate that saliva may be an alternative clinical material for both early diagnosis and prognosis of head and neck cancer (HNC). Therefore, we assessed salivary EBV DNA in oropharyngeal cancer patients (OPSCC). We observed that the EBV DNA level was higher in the group of EBV-positive OPSCC than in EBV-negative subjects and was also higher in more advanced clinical stages. In addition, the salivary EBV DNA load was positively correlated with the concentration of anti-EBV antibodies, MMP3, MMP9, and NF-κB. The ROC analysis confirmed the diagnostic accuracy of salivary EBV DNA load. Our preliminary results indicate the usefulness of determining EBV DNA load in saliva as a non-invasive prognostic biomarker in EBV-positive oropharyngeal cancer, but confirmation in larger cohort studies is required.

## 1. Introduction

Cancer is a serious challenge to modern medicine, both from clinical and epidemiological points of view. High morbidity and mortality rates provide an impetus to search for new diagnostic and therapeutic methods. In 2022, 20 million newly diagnosed cases of cancer and 9.7 million deaths from this cause were registered worldwide. It is expected that by 2050 this number will reach 35 million [1].

Head and neck cancer (HNC) accounts for 4.5% of all cancer diagnoses worldwide and is an important public health problem [2]. The most frequent histological type in this location is squamous cell carcinoma originating from the epithelium of the oral cavity, pharynx, or larynx [3].

In the Asian population, especially in China, NPC is the most common disease, hence most research focuses on this cancer [4]. In contrast, in Poland, oropharyngeal cancers predominate among head and neck cancers. The oropharynx and nasopharynx are distinct yet adjacent anatomical regions. According to data registered by Globocan, in 2022, 1699 new cases of oropharyngeal cancer were recorded in Poland, and only 210 cases of nasopharyngeal cancer [1].

The incidence of oropharyngeal squamous cell carcinoma (OPSCC) is increasing, which is mainly related to HPV infection. The relationship between EBV infection and the development of nasopharyngeal cancer has already been proven. There are few articles in the scientific literature regarding EBV-related oropharyngeal cancer [5,6]. Ruuskanen et al. [7] found that approximately 75% of Finnish patients with NPC are associated with EBV.

Epstein–Barr virus (EBV) (human herpes virus 4 (HHV-4), family *Orthoherpesviridae*, subfamily *Gammaherpesvirinae*, genus *Lymphocryptovirus*) is widely distributed in the human population [8,9,10]. It was discovered in 1964 by the following three scientists: Anthony Epstein, Yvonne Barr, and Burt Achong, and after many tedious laboratory and clinical tests, it was recognized as a virus with oncogenic potential. Many different researchers found that EBV infection was strongly associated with the development and/or progression of cancers originating from both B cells (Burkitt’s lymphoma, Hodgkin’s lymphoma) and epithelial cells (gastric cancer, NPC, breast cancer, thyroid cancer, salivary gland cancer, liver and bile duct cancer) [11,12,13,14,15,16,17]. For this reason, in 1997, EBV was classified as a group I carcinogen by the International Agency for Research on Cancer [18].

Although 60 years have passed since the discovery of the Epstein–Barr virus (EBV), its role in oncogenesis is not yet fully understood. EBV causes a wide range of diseases, from infectious mononucleosis to cancers. Many of these diseases are now treatable. New therapeutic methods are also being developed to specifically target EBV [19]. The ability of the EBV to establish a latent state makes eradication of the virus from the infected organism difficult, and the virus may remain in the latent phase of infection throughout a person’s life [20].

EBV transmitted through saliva initially infects B cells and oropharyngeal epithelial cells [8,15,19]. EBV infection of oral epithelial cells most often leads to lytic replication of the virus. Therefore, oral EBV DNA load may indicate not only EBV lytic replication activity but also potentially correlate with EBV-related disease progression. While searching for new markers with diagnostic and/or prognostic significance in HNCs, especially NPC, many researchers emphasize the importance of EBV viremia [21,22]. Numerous studies conducted in China, where this cancer is common, have shown that EBV tests can identify people in the early stages of the disease, before clinical symptoms develop, and can even detect people at high risk.

Saliva, an easily accessible, non-invasive clinical material, contains various molecules (proteins, peptides, nucleic acids, electrolytes, hormones) that may be useful diagnostic or prognostic biomarkers [23].

Many studies indicate that serological assessment of the level of anti-EBV antibodies is important in the early detection of NPC [24,25,26]. However, it is also important to assess the EBV DNA load [26,27,28,29].

Inspired by the results of the authors mentioned, we decided to evaluate EBV load in saliva in EBV-positive oropharyngeal cancer patients. For this purpose, the relationship between salivary EBV DNA load, histological differentiation (grading), and TN classification was analyzed. Moreover, we wanted to check the possible correlation between salivary EBV DNA load and serum level of selected biomarkers, i.e., metalloproteinases MMP3 and MMP9 and nuclear factor kappa NF-κB.

## 2. Materials and Methods

### 2.1. Basic Description of the Studied Groups

The study consisted of a total of 110 patients (86.2% man) with newly diagnosed and histopathologically confirmed squamous cell carcinoma of the oropharynx (OPSCC), hospitalized at the Department of Otolaryngology, Head and Neck Cancer, University of Technology and Humanities in Radom, Poland. Tumor tissue samples were collected during surgery. All clinical samples were examined by a pathologist—histopathological evaluation (grading, TNM) and Epstein–Barr virus-encoded small RNA transcripts (EBER)—in situ hybridization (ISH), and p16 HPV—immunohistochemical screening test for HPV. All patients with co-infection with EBV/HPV were excluded from the study.

So, for the presented research, we received 58 clinical samples where EBV was detected (hereinafter referred to as EBV-positive) and 52 where EBV was not detected (hereinafter referred to as EBV-negative). All patients had not received radiotherapy or chemotherapy before.

The majority of patients were men (86.2%). Two age groups were distinguished, i.e., 50–59 (54.7; SD = 2.6) and 60–79 (68.5; SD = 5.5).

Among the studied patients, most cases were diagnosed as G2—51.7%, T2—46.6%, and N0—39.7%. Advanced stages were found less frequently, i.e., G3—15.5%, T4—13.7%, and N3—17.2%. The basic demographic and clinical characteristics of the patients are presented in Table 1.

OPSCC patients tested negative for HIV, HBV, HCV, Parvovirus B19, HSV, and CMV infection. No anti-EBV IgM antibodies were found in any of the examined subjects. Excluding recent infections is important because it avoids interference with the analyzed parameters. Infections with the pathogens mentioned above may cause false-positive results.

During histopathological examination, an experienced pathologist determined the classification of tumors, nodes, and metastases (TNM) in accordance with the eighth edition of the TNM classification of HNCs [30,31,32]. Histological evaluation was performed according to the criteria of the World Health Organization, i.e., well differentiated (G1), moderately differentiated (G2), and poorly differentiated (G3) [33].

### 2.2. Collecting Clinical Samples

Saliva and serum samples collected from the patients with oropharyngeal cancer described above were used for research purposes.

#### 2.2.1. Collecting Saliva Samples

About 5 mL of unstimulated whole saliva (before surgery) was collected. All subjects were advised not to eat or drink anything 30 min before sample collection. Then, each patient was instructed to rinse their mouth with 10 mL of physiological saline (0.9% NaCl). After brief instructions from the nurse, all patients collected saliva themselves by spitting into a sterile tube. The saliva samples were centrifuged at 1500× *g* rpm at room temperature for 10 min, diluted (1:1) in PBS, and frozen at −80 °C until DNA extraction. Before DNA extraction, the saliva was thawed and centrifuged (3000× *g*, 10 min, 4 °C) to remove cellular debris and other impurities.

#### 2.2.2. Serum Collection

Both patient groups’ venous blood samples (collected according to hospital standards) were centrifuged at 1500× *g* rpm for 15 min at room temperature, and the serum was collected and frozen at −80 °C until analysis.

### 2.3. EBV DNA Detection in Saliva

DNA was extracted from saliva using the QIAampDNA Mini Kit (Qiagen, Hilden, Germany) according to the manufacturer’s protocol. The quality of the extracted DNA was then assessed through the implementation of the β-globin assay, which was utilized to ensure the integrity of the DNA and to identify the presence of any potential PCR inhibitors. Purified DNA was quantified using an Epoch Microplate Spectrophotometer (BioTek Instruments Inc., Vinooski, VT, USA).

Then, EBV DNA was amplified using the Gene Proof EBV diagnostic kit (GeneProof a.s. Vídeňská 101/119, Brno, Czech Republic) according to the manufacturer’s protocol (real-time qPCR). The EBV DNA copy number was assessed using the ISEX variant of the EBV PCR kit (GeneProof, Brno, Czech Republic). All samples were analyzed in duplicate with DNA elution buffer as an additional negative control. Amplification of the specific DNA sequence for EBNA1 was performed using LightCycler 2.0 software version 4.1 (Roche Applied Science System, Penzberg, Germany). The quantitative result of EBV DNA in saliva was given in copies/mL according to the manufacturer’s instructions. Results were normalized for DNA isolation efficiency, and the detection limit in saliva was 100 EBV DNA copies/mL.

### 2.4. Serological Methods Used in the Research

The level of anti-EBV antibodies and the concentrations of metalloproteinases MMP 3 and MMP 9, as well as NF-κB were determined using serological methods.

The level of anti-EBV antibodies of the IgA (Cat. No EBV IgA EBAMA96), IgM (Cat. No EBV IgM EBMMA96), and IgG (Cat. No EBV IgG EBGMA96) classes were determined with a commercially available Microblot-Array test (TestLine Clinical Diagnostics Ltd., Brno, Czech Republic). This test contains a combination of specific EBV antigens, i.e., EBNA1, EBNA2, VCA, EA, Rta, ZEBRA (Zta), gp85, gp350, and latent membrane protein 1 (LMP1). The occurrence of a reaction with at least one antigen, EBNA1 or VCA, is considered a positive result. The results are given in U/mL. Negative results are below 185 U/mL, borderline results are 185–210 U/mL, and positive results are above 210 U/mL. The test results were read using a Microblot-Array reader and software version 2.0.4.

Serum MMP levels were determined using the ELISA Kit for Matrix Metalloproteinase 3 (MMP3) (Cat No SE-A101Hu) and the ELISA Kit for Matrix Metalloproteinase 9 (MMP9) (Cat No SE-A553Hu) (Cloud-Clone Corp., Katy, TX, USA). The minimum detectable dose for MMP3 is usually less than 13.1 pg/mL (detection range 31.2–2000 pg/mL), while for MMP9 the minimum detectable dose is usually less than 0.055 ng/mL (detection range 0.156–10 ng/mL).

Serum NF-κB levels were determined using a commercially available ELISA Assay Kit for Nuclear Factor Kappa B (NF-κB)—Cat. No SE-B824Hu (Cloud-Clone Corp.; CCC, USA). The minimum detectable concentration of NF-κB is less than 0.059 ng/mL (detection range 0.156–10 ng/mL).

All analyzed serum samples were tested in duplicates. The antibodies concentrations, as well as MMP3, MMP9, and NF-κB, were read from the standard curve (OD 450 nm) and presented as continuous values (U/mL).

### 2.5. Statistical Analysis

Tibco Statistica 13.3 (StatSoft, Kraków, Poland) and GraphPad Prism version 10.4.0 (San Diego, CA, USA) were used in the statistical analysis of the research results.

The Shapiro–Wilk test was used to check the normal distribution of continuous variables. The relationship between sociodemographic characteristics was assessed using the Pearson chi-square test. In order to compare EBV DNA load according to grading and T and N classification, the Mann–Whitney Test was carried out. The correlation between salivary EBV DNA load, anti-EBV antibodies, and selected biomarkers was assessed using the Spearman rank test. The significance level was adjusted using the Bonferroni correction for multiple comparisons. The diagnostic accuracy of salivary EBV DNA load was determined by receiver operating characteristic (ROC) curve analysis. The ROC curve shows a comparison of the sensitivity and specificity of the tested salivary EBV DNA in EBV-positive and EBV-negative patients.

## 3. Results

### 3.1. Comparison of Salivary EBV DNA Load in EBV-Positive and EBV-Negative OPSCC Patients

**Figure 1:**
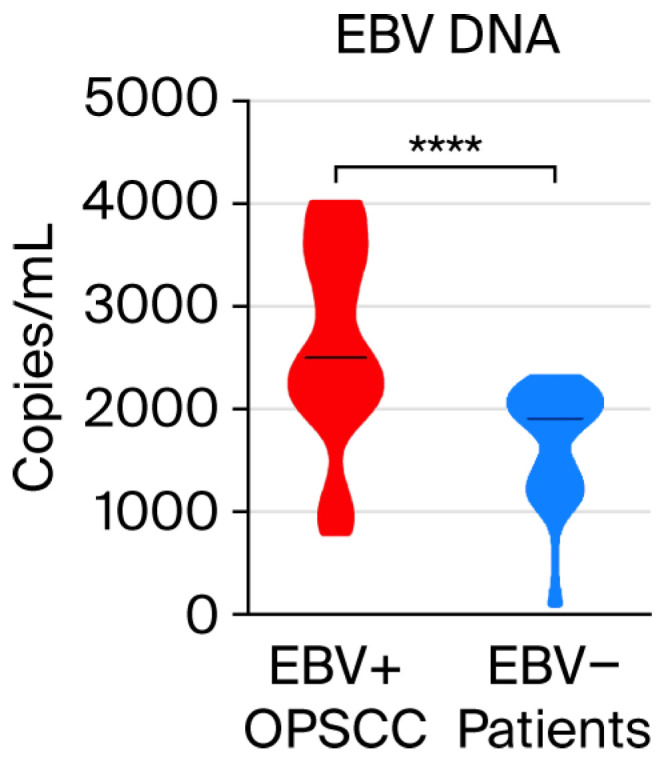
Comparison of salivary EBV DNA load in EBV-positive and EBV-negative oropharyngeal patients. **** *p* < 0.0001.

First, the presence of EBV DNA in the saliva of 52 EBV-negative OPSCC patients was examined. Out of 52 people, EBV DNA was detected in 43 cases, which constitutes 82.7%. EBV DNA levels in the saliva of both patient groups were then compared (Figure 1). Detailed data are provided in the Supplementary Materials (Table S1). We found that the level of EBV DNA in the EBV-negative OPSCC patients was significantly lower than in EBV-positive OPSCC patients (1900 vs. 2500 copies/mL; *p* < 0.0001).

### 3.2. Assessment of EBV DNA Load in the Saliva of Patients with Oropharyngeal Cancer Depending on the Grade (G1–G3) and TN Classification

**Figure 2:**
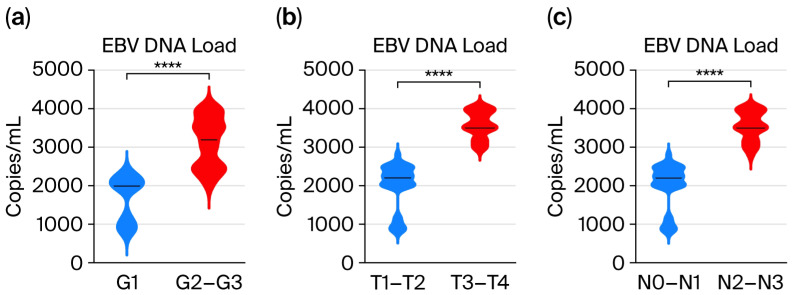
Salivary EBV DNA load (copies/mL) in EBV-positive OPSCC: (**a**) by grading, (**b**) by T stage, and (**c**) by N stage; Mann–Whitney Test; **** *p* < 0.0001.

Therefore, it seemed advisable to assess the level of EBV DNA depending on the degree of tumor differentiation and T and N features (Figure 2). The exact values are shown in Table S2 (Supplementary Materials). The obtained results indicate a relationship between the level of viral load and clinicopathological features, i.e., in more advanced clinical stages of this cancer, a higher level of EBV DNA was observed.

### 3.3. Correlation Analysis Between the EBV Load in Saliva and the Titer of Anti-EBV Antibodies in the Serum of Oropharyngeal Cancer Patients

**Figure 3:**
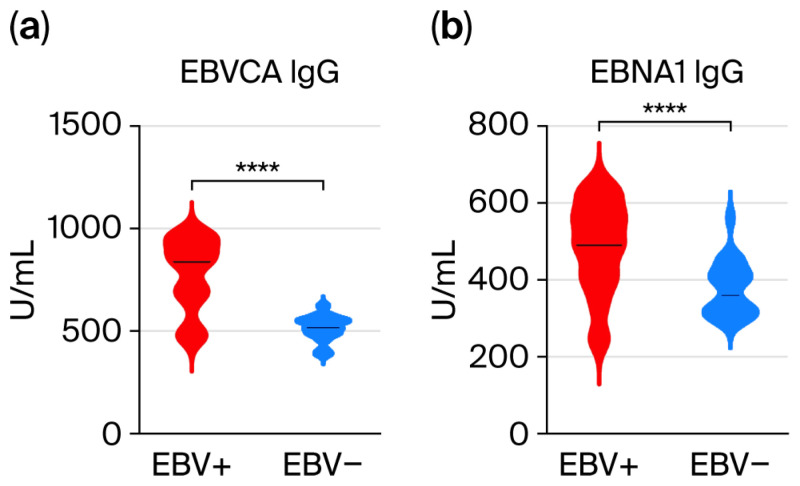
Levels of anti-EBVCA (**a**) and anti-EBNA1 (**b**) antibodies in IgG class in EBV-positive and EBV-negative OPSCC patients. **** *p* < 0.0001.

First, we assessed the titer of anti-EBV antibodies in the studied groups of patients (Supplementary Materials Table S3). The obtained results indicate a significantly higher titer of EBVCA (777.1 Vs. 514.6 U/mL) and EBNA1 (487.0 Vs. 375.6 U/mL) antibodies in the IgG class in EBV-positive patients than in the EBV-negative group (Figure 3).

However, neither EBVCA IgA nor EBNA1 IgA antibodies were detected in EBV-negative patients. Other antibodies such as anti-EA, anti-Zta, and anti-LMP1 were detected only in the serum of EBV-positive patients (Supplementary Materials Table S3).

**Figure 4:**
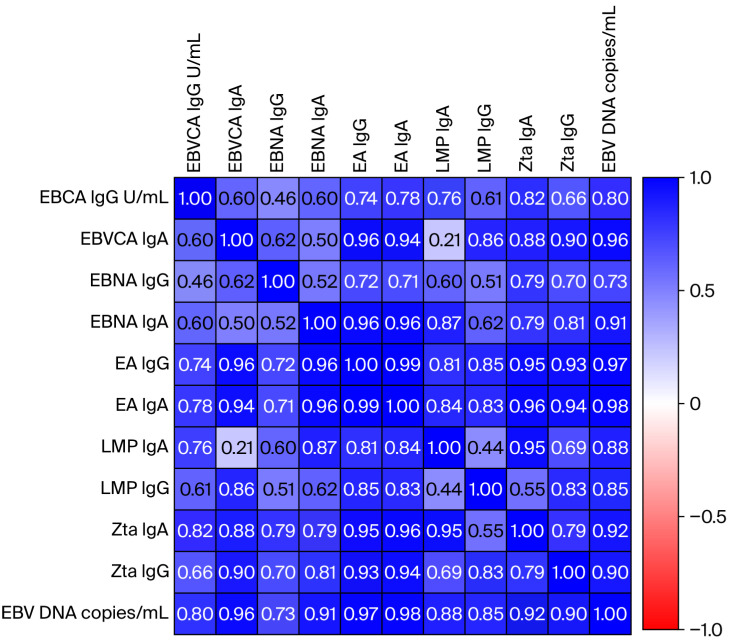
Correlation between salivary EBV DNA load and anti-EBV antibodies in EBV-related OPSCC patients. Spearman rank coefficients are presented as color intensities. The closer R is to +1 or −1, the stronger the correlation. A perfect positive correlation is +1 (blue), and a perfect negative correlation is −1 (red). The significance level was adjusted using the Bonferroni correction for multiple comparisons.

Then, the relationship between the level of individual anti-EBV antibodies and the EBV DNA load in saliva was analyzed to check for a possible correlation (Figure 4). After statistical analysis using multiple linear regression, a high correlation was demonstrated, which was graphically presented on a heat map. The exact values of the Spearman rank coefficient for individual types of anti-EBV antibodies are presented in Table S4 (Supplementary Materials). A highly significant relationship was found between EBV load and the titer of EBNA1, EBVCA, EA, Zta, and LMP1 antibodies, both in the IgA and IgG classes.

### 3.4. Receiver Operating Characteristic (ROC) Curve Analysis to Determine the Diagnostic Accuracy of Salivary EBV DNA Load in EBV-Positive OPSCC Patients Compared to EBV-Negative Subjects

**Figure 5:**
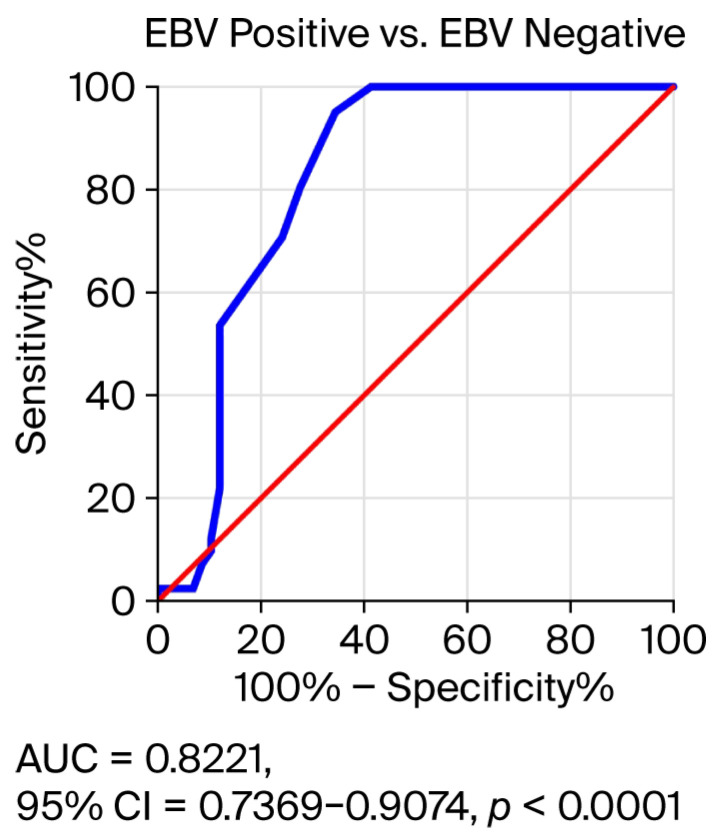
Receiver operating characteristic (ROC) analysis for EBV DNA load in saliva of EBV-related OPSCC patients. Blue line—salivary EBV DNA load (copies/mL); red line—reference line.

At this stage of the research, we checked the accuracy of determining the EBV DNA load in saliva, i.e., whether it can be a useful diagnostic and/or prognostic biomarker in OPSCC (Figure 5). The results confirmed this hypothesis because AUC = 0.8221; Std. Error 0.04350; 95% CI = 0.7369–0.9074, *p* < 0.0001. Therefore, the EBV DNA load in saliva may be a good marker in the prognosis of EBV-positive oropharyngeal cancer.

### 3.5. Correlation Analysis Between the EBV Load in Saliva and the Level of Selected Biomarkers in the Serum of Oropharyngeal Cancer Patients

**Figure 6:**
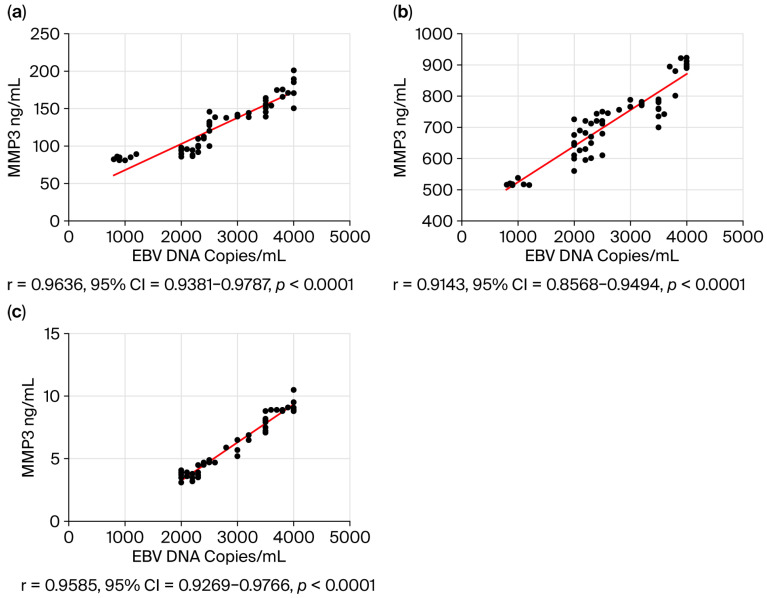
Correlation between salivary EBV DNA load and selected biomarkers in EBV-positive OPSCC patients: (**a**) MMP3, (**b**) MMP9, and (**c**) NFκB. The significance level was adjusted using the Bonferroni correction for multiple comparisons.

In the final step of our study, we assessed the correlation between selected serum biomarker levels and salivary EBV DNA load in patients with EBV-positive OPSCC (Figure 6). In the current analysis we included metalloproteinases, i.e., MMP 3 (Figure 6a) and MMP 9 (Figure 6b), as well as NFκB (Figure 6c). The obtained results showed that the higher the EBV DNA load in saliva, the higher the concentration of MMP3, MMP9, and NFκB in the serum.

## 4. Discussion

Viruses are responsible for 10–15% of human cancers worldwide [34,35]. Oncoviruses are therefore the main target in diagnosis, therapy, and prevention.

The discovery of EBV over 60 years ago was a breakthrough in understanding the development of many cancers, including head and neck cancers. Many processes have already been described, but many remain to be explained.

As mentioned in the Introduction, the vast majority of studies concern NPC, a cancer endemic in China. Researchers are looking for new biomarkers that would allow for early diagnosis [36]. Our experiences fit into this direction of research.

Many authors use saliva as a tool for early diagnosis of oral cancer [22,37,38,39,40,41]. Recently, researchers have been paying great attention to the EBV DNA load, especially in saliva, which may constitute an alternative clinical material not only for early diagnosis but also for prognosis of this disease [4]. However, similar studies regarding EBV-related OPSCC have not been found in the available literature. Therefore, we wanted to check what this problem looks like in the case of oropharyngeal cancer.

Even though we detected EBV DNA in saliva in a high percentage, i.e., 82.7%, in the EBV-negative patients, the EBV load was significantly lower compared to the EBV-positive group (1900 vs. 2500 copies/mL; *p* < 0.0001).

Our analysis showed a relationship between the level of EBV DNA and the clinical features of OPSCC. Among the patients studied, higher levels of EBV DNA were observed in more advanced clinical stages of cancer. We observed that OPSCC patients with advanced T stage (T3–T4) had a significantly higher EBV DNA load. Similar results were obtained by He et al. [21] analyzing a group of patients with NPC. The ROC analysis showed a significant prognostic value of EBV DNA in saliva.

According to the above-mentioned authors, oral EBV DNA load, a biomarker that is an indicator of EBV lytic replication, has not yet been analyzed in the context of its prognostic value in NPC patients. Furthermore, a positive association between T stage and oropharyngeal EBV DNA levels has been reported when examining plasma or nasopharyngeal brush swabs [27,42,43]. In turn, other authors stated that although EBV DNA is useful in the diagnosis of NPC, the content of EBV DNA in saliva is not important in the diagnosis of NPC [44]. These authors note, however, that due to the unique geographical distribution, their findings may have limited generalizability to other regions. However, the combined assessment of EBV DNA load in plasma and EBV antibody titers is a useful tool in the diagnosis, prognosis, and treatment of NPC [44,45].

Interesting results were presented by Zheng et al. [46], examining EBV DNA load and EBV DNA methylation in saliva, oropharyngeal swabs, oral swab, and mouthwash samples of NPC. In their previous study, they presented a saliva biopsy to detect EBV DNA methylation, demonstrating the possibility of using oral samples in the diagnosis of NPC [47]. These authors observed an increase in EBV methylation in all types of samples from NPC patients. However, the authors emphasize that EBV DNA methylation in saliva and throat swabs had better diagnostic performance, which indicates that they may constitute potential biomarkers of NPC. Therefore, further studies of EBV DNA load and EBV DNA methylation in these individuals appear warranted to determine both the best sampling method and the best diagnostic value.

Numerous studies prove that there is a strong relationship between lytic replication and cancer development. EBV lytic replication is a complex process that occurs at the molecular level and involves a variety of host cell proteins. So, it can be assumed that lytic replication would increase virus production and, consequently, the number of infected cells that could transform [48].

Due to increased EBV replication, there is an increase in the titer of specific antibodies as well as EBV load in saliva. Our research showed a high correlation between EBV DNA load in saliva and the titer of specific anti-EBV antibodies.

In the routine diagnosis of EBV infections, serological methods detecting specific antibodies against the capsid antigen (EBVCA), nuclear antigen 1 (EBNA1), and early antigen (EA) are used [49,50]. As numerous studies have shown, higher levels of anti-EBVCA, anti-EA, and anti-EBNA antibodies are found in the serum of NPC patients [51,52,53].

In the initial phase of lytic EBV replication, the Zta protein is synthesized, which is the product of the BZLF1 gene [54]. It is of great importance in the diagnosis of NPC, apart from VCA-IgA, EBNA1-IgA, and Rta-IgG antibodies.

Our analysis showed high titers of EBVCA, EBNA, EA, and Zta antibodies in both IgA and IgG classes in the serum of EBV-positive OPSCC patients. Many authors have observed high titers of anti-Zta IgG antibodies in NPC patients, including those negative for anti-EBVCA and anti-EA, both IgG and IgA [55,56,57,58]. The last-mentioned author [54] conducted an interesting, systematic assessment of the diagnostic value of serum anti-Zta antibodies in patients with NPC, emphasizing that the detection of VCA-IgA, EBNA1-IgA, and Rta-IgG is highly accurate in the early diagnosis of NPC. Our analysis also showed high titers of EBVCA, EBNA, EA, and Zta antibodies in both IgA and IgG classes in the serum of EBV-positive OPSCC patients.

Both genetic and epigenetic factors have a significant impact on changes in the tumor microenvironment (TME). Many of these changes are controlled by metalloproteinases (MMPs). Disturbed expression of MMPs leads to the development and progression of various cancers [58]. Many researchers point to a positive correlation of MMP3 and MMP9 with the clinical stage in patients with NPC [59,60,61]. As shown by Li et al. [62], simultaneous assessment of MMP 3 activity and EBV antibodies may be a valuable marker in the diagnosis of NPC. However, other studies have shown that Zta—the lytic transactivator of the Epstein–Barr virus (EBV)—increases the expression of MMP 3 and MMP 9 [63].

In turn, nuclear factor beta (NF-κB) controls the expression of over 500 genes involved in various processes, such as inflammatory and immune responses (innate and adaptive) as well as cell proliferation, differentiation, and survival [64,65,66,67]. The role of the NF-κB pathway in the development of NPC has been described in detail in many publications [68,69,70,71].

MMP 3, MMP 9, and NF-κB participate in different signaling pathways in the tumor microenvironment. Therefore, in the current study we wanted to answer the question of whether there is a relationship between the level of EBV DNA in saliva and the concentration of the above-mentioned biomarkers in serum. Using the Spearman rank test, we observed a high correlation between EBV DNA load in saliva and the level of these markers in serum, i.e., a higher EBV DNA load was accompanied by a higher level of MMP 3, MMP9, and NF-κB.

The role of EBV in the pathogenesis of many human diseases highlights the need for an effective vaccine [72,73]. Unfortunately, there is no EBV vaccine, neither prophylactic nor therapeutic. Despite many trials of various vaccines such as subunit vaccines, epitope vaccines, DNA vaccines, protein vaccines, vector vaccines, and virus-like particles (VLPs), none of them provides sufficient protection. It is widely recognized that vaccination against EBV could change the landscape of EBV-related disease, including cancers.

### Limitations of Own Research

Due to the fact that this study is a continuation of earlier research, we had a small number of individual samples, which limited the number of parameters determined. The small size of the research group results from the low incidence of oropharyngeal cancer in our region, which causes great difficulties in collecting material. The resulting small numbers in the subgroups forced us to combine the groups and analyze them together, i.e., grading as G1 and G2-G3; T (tumor size) as T1–T2 and T3–T4; and N (lymph node involvement) as N0–N1 and N2–N3.

Over the past three to four decades, there has been a significant increase in the incidence of oropharyngeal squamous cell carcinoma (OPSCC). Although it is most often associated with HPV infection, EBV plays an equally important role. EBV infection has been detected in 25.9–82.5% of OSCCs, and HPV/EBV co-infection prevalence in OSCCs varies between 6.5% and 37.5% [74]. Carpén et al. [5] observed that EBV-positive OPSCC cases (expressing EBER) had poorer survival compared to EBV-negative OPSCC. Therefore, these authors suggest that this novel observation may identify a new subgroup of patients with non-HPV-related OPSCC.

As Weixing Liu [75] wrote in one of his publications, “the accuracy of diagnosis is not influenced by sample size or ethnicity. Given the small number of studies in non-Asian populations, the results obtained require confirmation in another population”. Therefore, we believe that despite the above-mentioned limitations, our research seems fully justified.

According to Blanco et al. [76], both epidemiological and clinical evidence suggests that HR-HPV/EBV co-infection may play a significant role in the development of these cancers, which requires further research. In the future, it would be worth assessing how EBV/HPV co-infection affects EBV DNA load and other analyzed parameters.

Although the obtained results are promising, they concern only the studied group of patients from one clinical center. Therefore, in order to draw general conclusions, a much larger group of patients should be examined. Future studies should also include other types of oral samples, e.g., oral swabs or mouthwash samples of oropharynx to help select the most diagnostically useful clinical specimen.

## 5. Conclusions

EBV DNA levels are determined in plasma and, more recently, also in other clinical samples. Some scientists pay attention to saliva as a valuable source of potential biomarkers.

Our research shows that higher concentrations of EBV DNA were observed in more advanced clinical stages in EBV-positive OPSCC, therefore it seems that it may have a prognostic rather than diagnostic significance. In addition, we observed a high correlation between salivary EBV DNA load and the concentration of selected markers in serum, such as MMP 3, MMP 9, and NF-κB. The results presented are preliminary and require confirmation in larger cohort studies.

Nevertheless, we hope that our results will provide new insight into the possibility of using salivary EBV DNA load as a non-invasive, easily accessible biomarker in monitoring progression and possibly response to therapy of EBV-related OPSCC.

## Figures and Tables

**Table 1:** Baseline characteristics of oropharyngeal patients.

		EBV				*p*	Total Patients	
		Positive		Negative				
		N	%	N	%		N = 110	%
Sex	Female	8	13.8	7	13.5	0.9999	15	13.8
	Male	50	86.2	45	86.5		95	86.2
Age	50–59	27	46.6	24	46.2	0.1116	59	53.4
	60–79	31	53.4	28	53.8		51	46.6
Place of residence	Urban	41	70.7	36	69.2	0.1667	77	70.7
	Rural	17	29.3	16	30.8		33	29.3
Smoking	≤10 *	28	48.3	25	48.1	0.8427	53	48.3
	>10	10	17.2	10	19.2		20	18.2
	No	20	34.5	17	32.7		37	34.5
Alcohol abuse	≤10 **	18	31.1	15	28.8	0.9834	53	48.3
	>10	10	17.2	10	19.3			
	No	30	51.7	27	51.9		57	51.7
G	G1	19	32.8	17	32.7	0.9997		
	G2	30	51.7	27	51.9			
	G3	9	15.5	8	15.4			
T	T1	7	12.1	8	15.4	0.9505		
	T2	27	46.6	22	42.3			
	T3	16	27.6	15	28.8			
	T4	8	13.7	7	12.1			
	N0	23	39.7	22	42.3	0.9844		
N	N1	11	19.0	10	19.2			
	N2	14	24.1	11	21.2			
	N3	10	17.2	9	17.3			
M	M0	58	100.0	52	100.0			

Pearson’s chi-square test; * packs/week; ** drink/week.

## Data Availability

Due to privacy and ethical concerns, the data used in this study are available from the corresponding author upon reasonable request.

## Supplementary Materials

The following supporting information can be downloaded at: https://www.mdpi.com/article/10.3390/v17111523/s1, Table S1: Salivary EBV DNA load in oropharyngeal cancer patients EBV positive and EBV negative; Table S2: Salivary EBV DNA load (copies/mL) by grading (G) and T, N Classification among EBV Positive Oropharyngeal Cancer Patients; Table S3: Serum anti- EBV antibody levels in EBV positive and EBV negative OPSCC patients; Table S4: Correlation between the salivary EBV DNA load and anti-EBV antibodies in EBV-related OPSCC patients; Table S5: MMP 3 and MMP 9 concentration in the serum EBV positive and EBV negative OPSCC patients; Table S6: NF-kB concentration in the serum EBV positive and EBV negative OPSCC patients (ng/mL).

## Abbreviations

The following abbreviations are used in this manuscript:

HNC	Head and neck cancer
NPC	Nasopharyngeal carcinoma
OPSCC	Oropharyngeal squamous cell carcinoma
EBV	Epstein–Barr virus
NF-κB	Nuclear factor kappa
MMP3	Metalloproteinase 3
MMP9	Metalloproteinase 9
LMP	Latent membrane protein
VLPs	Virus-like particles
