# Supplementary material for: Epstein–Barr Virus Load in the Saliva of Patients with Oropharyngeal Cancer—Could It Have Prognostic Significance?
Source: Viruses. 2025 Nov 20;17(11):1523. doi: 10.3390/v17111523 (PMC12656936; doi:10.3390/v17111523)
Supplement: Supplementary file 1 [file viruses-17-01523-s001.zip › viruses-3997384-supplementary.pdf]

Supplementary Materials

Table S1. Salivary EBV DNA load in oropharyngeal cancer patients EBV positive and EBV negative.

OPSCC patients	Median	Min-max	95% CI of median	*p* value
EBV negative	1900	100 – 2300	97.25	
EBV positive	2500	800 – 4000	95.21	< 0.0001*

Table S2. Salivary EBV DNA load (copies/mL) by grading (G) and T, N Classification among EBV Positive Oropharyngeal Cancer Patients.

Parameter	Mean	Min - Max	SD	*p* value
G1	1687	800 – 2300	580.4	
G2-G3	3128	2000 – 4000	649.3	< 0.0001*
T1 – T2	2005	800 – 2800	576.5	<0.0001*
T3 – T4	3579	3000 – 4000	346.4	
N0 – N1	2005	800 – 2800	576.5	< 0.0001*
N2 – N 3	3548	2800 – 4000	373.2	

*Statistically significant; Mann-Whitney Test.

Table S3. Serum anti- EBV antibody levels in EBV positive and EBV negative OPSCC patients

Antibodies U/mL	Group	Mean	Median	Min	Max	SD	*p* value
EBVCA IgG	EBV+	777.1	837.5	448.9	980.9	173.9	<0.0001*
	EBV -	514.6	515.4	385.2	622.3	60.4	
EBVCA IgA	EBV+	505.8	456.9	243.6	923.6	197.9	
	EBV -	-	-	-	-	-	
EBNA1 IgG	EBV +	487.0	490.9	230.4	655.1	117.3	<0.0001*
	EBV -	375.6	360.2	290.5	562.1	68.6	
EBNA1 IgA	EBV +	683.2	745.9	260.5	902.5	230.7	
	EBV -	-	-	-	-	-	
EA IgG	EBV +	584.5	395.0	234.5	948.5	166.0	
	EBV -	-	-	-	-	-	
EA IgA	EBV +	551.9	517.0	349.9	950.5	148.3	
	EBV -	-	-	-	-	-	
Zta IgA	EBV +	664.8	657.8	274.8	989.7	240.6	
	EBV -	-	-	-	-	-	
Zta IgG	EBV +	656.7	641.6	260.5	985.6	235.4	
	EBV -	-	-	-	-	-	
LMP1 IgA	EBV +	467.8	501.2	240.5	610.8	128.5	
	EBV -	-	-	-	-	-	
LMP1 IgG	EBV +	490.0	529.2	260.8	675.9	125.1	
	EBV -	-	-	-	-	-	

*Statistically significant; Mann-Whitney Test

Table S4. Correlation between the salivary EBV DNA load and anti-EBV antibodies in EBV-related OPSCC patients.

**Antibody U/mL**	**Spearman r**	**95% CI of rs**	***p value***
EBNA IgA	0.907	0.7767 – 0.9632	< 0.0001*
EBNA IgG	0.727	0.5475 – 0.8427	< 0.0001*
EBVCA IgA	0.959	0.8909 - 0.9848	0.0001*
EBVCA IgG	0.800	0.6701 - 0.8826	0.0001*
LMP1 IgA	0.876	0.7070 - 0.7955	0.0001*
LMP1 IgG	0.849	0.7116 - 0.9239	< 0.0001*
Zta IgA	0.916	0.8331 - 0.9591	< 0.0001*
Zta IgG	0.902	0.7990 - 0.9538	< 0.0001*
EA IgA	0.976	0.9580 – 0.9859	<0.0001*
EA IgG	0.974	0.9543 - 0.9847	<0.0001*

*Statistically significant.

Table S5. MMP 3 and MMP 9 concentration in the serum EBV positive and EBV negative OPSCC patients.

MMP (ng/mL)	Group	Mean	Minimum	Maximum	SD	*p* Value
MMP 3	EBV+	125.8	81.2	201.3	35.1	
	EBV−	80.4	71.5	92.8	7.2	<0.0001*
MMP 9	EBV+	715.7	515.5	923.4	115.3	
	EBV−	379.2	300.8	489.6	65.7	<0.0001*

*Statistically significant, Mann-Whitney Test

Table S6. NF-κB concentration in the serum EBV positive and EBV negative OPSCC patients (ng/mL)

Group	Mean	Minimum	Maximum	SD	*p* Value
EBV+	5.9	3.1	10.5	2.2	<0.0001*
EBV−	3.1	1.0	5.2	1.4	

*Statistically significant; Mann-Whitney Test
